# Fetal alcohol spectrum disorders prevention and clinical guidelines research - workshop report

**DOI:** 10.1186/s12919-024-00298-x

**Published:** 2024-08-07

**Authors:** Tracey Pérez Koehlmoos, Elizabeth Lee, Ilse Rivera, Jennifer Wisdahl, Katie Erdman, Tom Donaldson

**Affiliations:** 1 Center for Health Services Research, Uniformed Services University of the Health Sciences, Bethesda, USA; 2 Department of Pediatrics, Uniformed Services University of the Health Sciences, Bethesda, USA; 3 The Henry M. Jackson Foundation for the Advancement of Military Medicine, Inc., Bethesda, USA; 4 FASD United, Washington D.C., USA

**Keywords:** Fetal Alcohol Spectrum Disorders, FASD, Prenatal alcohol exposure, Military Health System

## Abstract

It is estimated that up to 1 in 20 people in the United States may have a fetal alcohol spectrum disorder (FASD), or the array of physical, cognitive, emotional, and social disorders caused by exposure to alcohol during prenatal development (May et al., JAMA 319:474–82, 2018). While this condition is present in a broad range of individuals and families, it has not previously been examined in the military community, where cultural factors including an increased prevalence of alcohol misuse may pose a unique set of challenges (Health.mil, Alcohol misuse, 2024).

The Uniformed Services University of the Health Sciences (USUHS), in conjunction with FASD United, hosted the second annual *Workshop on Fetal Alcohol Spectrum Disorders Prevention and Clinical Guidelines Research* on 20 September 2023 in Washington, DC. Organized as part of a four-year, federally-funded health services research initiative on FASD in the U.S. Department of Defense (DoD) Military Health System (MHS), the workshop provided a forum for exploring the initiative’s focus and progress; examining current knowledge and practice in the research and clinical spheres; and identifying potential strategies to further improve prevention, screening, diagnosis, interventions, and family support. Building off of the 2022 workshop that covered the state of the science surrounding prenatal alcohol exposure and FASD, the 2023 focused primarily on FASD and efforts aimed at identification and management (Koehlmoos et al., BMC Proc 17 Suppl 12:19, 2023). One hundred and thirty attendees from academia, healthcare, federal agencies, and patient advocacy organizations gathered to share research findings; learn from lived experiences; and discuss initiatives to advance research, screening, and services for at-risk pregnant women as well as families and caregivers supporting individuals with FASD.

## Background

In 2022, the Center for Health Services Research (CHSR) at USUHS, FASD United, and the Boston University School of Public Health embarked on a four-year, federally funded health services research initiative on FASD in the MHS. The FASD Prevention and Clinical Guidelines Research initiative supports investigations of the impact of FASD on families, working-age parents, and prospective parents in the military community. By shedding light on factors such as disease burden; population-level health outcomes; and care quality, efficiency, and access, the research findings will be used to develop medical and behavioral health interventions for individuals with FASD and their families. While the initiative focuses on members of the military and their families, the evidence base generated through this effort can also help to inform clinical practice guidelines for replication in civilian care systems.

The MHS is one of America’s largest and most complex health care institutions, providing direct health services and private sector care to approximately 9.6 million beneficiaries, including uniformed service members, military retirees, and their families. CHSR provides direct support services to DoD and the MHS, conducts research in support of MHS strategic goals and objectives, provides Health Services Research (HSR) training to students and faculty, and supports civilian HSR organizations. FASD United, the voice of the FASD community, aims to expand the capacity of FASD-informed diagnostic, medical, behavioral health, and non-clinical services; advocate for policy changes guaranteeing full inclusion in all systems of care and benefits programs for children and adults with FASD; and prevent prenatal exposure to alcohol and other harmful substances.

## Introduction

The Uniformed Services University of the Health Sciences (USUHS), in conjunction with FASD United, hosted the second annual *Workshop on Fetal Alcohol Spectrum Disorders Prevention and Clinical Guidelines Research* on 20 September 2023 in Washington, DC. One hundred and thirty attendees from academia, healthcare, federal agencies, and patient advocacy organizations gathered to review the latest research and discuss key issues surrounding FASD during the day-long workshop (see Appendix A for a list of attendees).issues surrounding FASD during the day-long.

The event was the second in a series of workshops held as part of the FASD Prevention and Clinical Guidelines Research initiative. The first workshop, held in 2022, provided an overview of the state of the science of research, programs, and clinical practice guidelines surrounding prenatal alcohol exposure and FASD [1]. The 2023 workshop detailed several of the initiative’s lines of effort, including assessments focused on community needs and population health, the use of electronic health records (EHRs) for predictive analytics to improve early diagnosis and patient outcomes, and the development of a telehealth hub-and-spoke model. USUHS’s external partners in this effort are FASD United, Boston University School of Public Health, and the Henry M. Jackson Foundation for the Advancement of Military Medicine (HJF); internal USUHS partners are the Center for Health Services Research (CHSR); the School of Medicine Departments of Preventive Medicine & Biostatistics, Clinical Psychology, Gynecologic Surgery and Obstetrics, Family Medicine, and Pediatrics; and the Graduate School of Nursing.

**Fig. 1:**
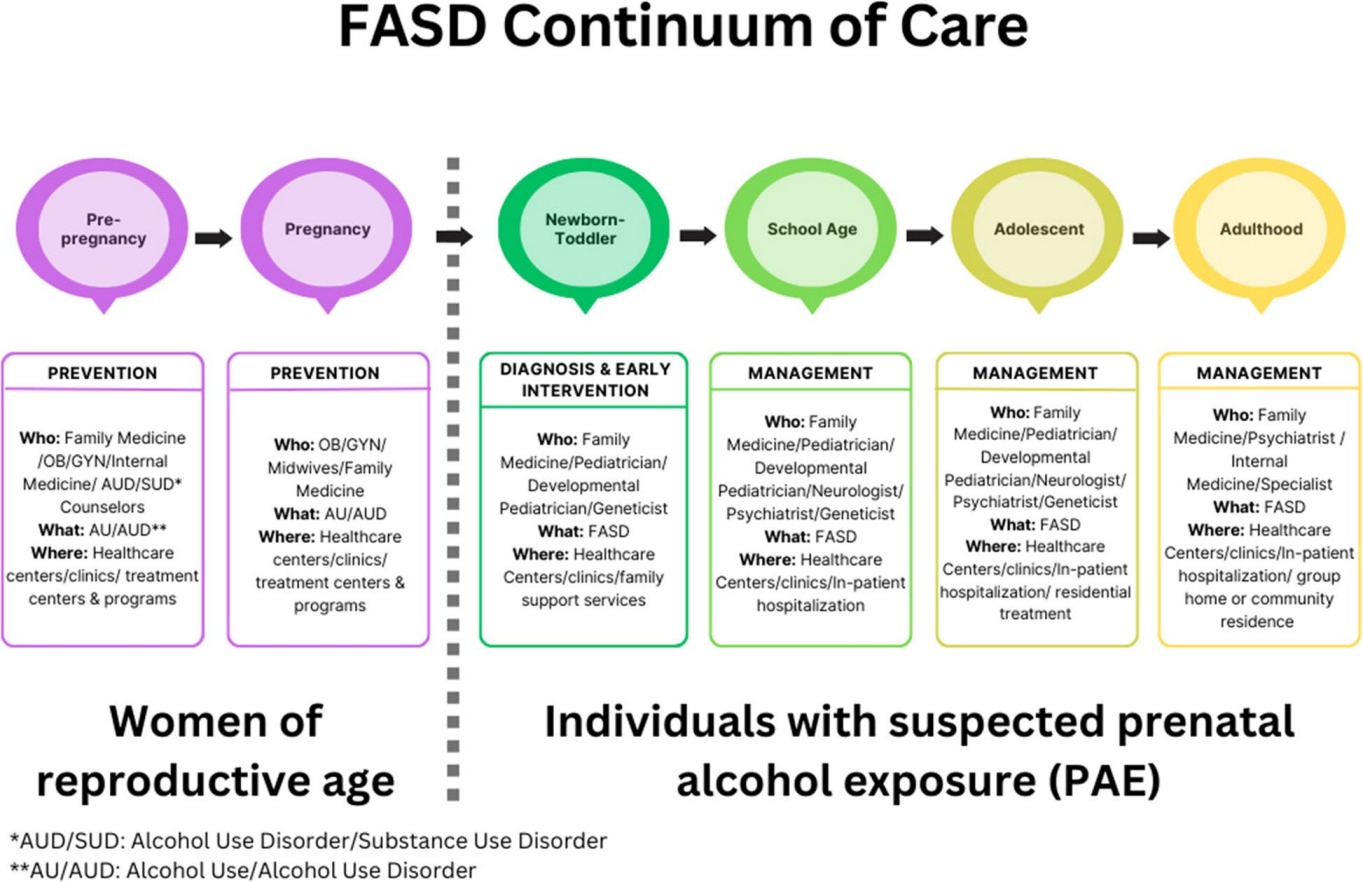
The FASD continuum of care

Tracey Pérez Koehlmoos, Director of the Center for Health Services Research at USUHS, and Tom Donaldson, FASD United President and CEO, provided welcoming comments. They noted that the increased attention and research into FASD has created a sense of optimism that the field is on the verge of important breakthroughs with the potential to improve the lives of individuals with FASD and their families. The workshop began with a presentation on building FASD-responsive systems of care. Drawing upon research findings and the lived experiences shared by individuals with FASD, participants then delved into opportunities to strengthen FASD clinical practices and guidelines and discussed risk factors, community needs, and the role of tools such as telehealth in improving diagnosis, care, and support. In the workshop’s closing session, representatives from federal agencies highlighted initiatives aimed at advancing research, surveillance, prevention, and care for individuals and families with FASD. Throughout the workshop, participants emphasized the importance of closing gaps and addressing needs along the entire continuum of care from pre-pregnancy prevention through the management of potential FASD impacts in childhood, adolescence, and adulthood, while recognizing the roles of different types of professionals, programs, and organizations across this spectrum (Fig. 1).

### State of the science: building FASD-responsive systems of care

FASD is a complex public health issue with myriad implications for individuals and families, clinicians and healthcare systems, and society more broadly. To set the stage for the workshop discussions, Christie L. M. Petrenko, University of Rochester and Mt. Hope Family Center, described how the body of knowledge arising from implementation science and effectiveness research can help to build and disseminate systems of care that appropriately recognize FASD; reduce associated stigmas; and leverage effective and synergistic measures for prevention, identification, and support. She also highlighted practical support tools and emerging innovations in FASD diagnosis and care, along with ways to influence change to better meet the needs of individuals and families living with FASD in the future.

### Learning from implementation science and effectiveness research

Implementation science and effectiveness research provide useful frameworks and insights for informing impactful health interventions. In a simplified implementation science framework, the first step is to identify the needed intervention, such as prenatal alcohol exposure (PAE) screening. Based on the identified needs, researchers develop and implement specific methods, such as training or EHR-embedded prompts to help providers conduct screenings. As part of this process, it is important to understand how the intervention is intended to work and what is needed to enable people or organizations to implement it. Finally, the outcomes of the intervention are evaluated to inform ongoing and future efforts [2].

In addition to informing and assessing particular interventions, implementation science offers models for successfully putting ideas into practice. For example, the co-model of behavior change states that successful implementation should include the capabilities, opportunities, resources, leadership support, and motivation to change clinicians’ behavior. In the FASD context, attending to these factors can help give clinicians the confidence to practice FASD-informed care and the knowledge that doing so will benefit their patients and themselves [3].

Effectiveness research can help to determine a method’s likelihood of success. This type of research has shed light on a variety of FASD prevention, identification, and support strategies. Familiar interventions such as public service announcements or warning labels may increase awareness, but there is little evidence of their impact on behavior. Instead, studies underscore the importance of considering an individual’s alcohol use, sexual activity, and contraceptive use when discussing PAE prevention, [4] as well as the benefits of involving patients’ partners and social support networks in PAE discussions. Effective screening strategies include asking about drinking prior to pregnancy recognition and embedding PAE questions into discussions about broader health topics. Overall, research suggests that discussions around alcohol and PAE should be conducted across multiple medical settings, involve relatives and friends as well as pregnant people, and be carefully documented to aid future diagnoses, Petrenko said.

In designing and implementing effective interventions, research also points to the need to address the potential for clinician bias, which can negatively impact support programs, resources, and patients’ sense of self-worth [5]. To avoid perpetuating the stigma that has often been associated with PAE and FASD, it is important for clinicians to remove potentially judgmental language or images from screening practices and materials, for example by using terms such as “prenatal alcohol exposure” instead of “maternal drinking.”

There are now several diagnostic tools that have been shown to be effective for identifying FASD. Each tool has slight variations that affect research, clinical practice, access to services, and reimbursements; the International Consensus Committee on Research Classification of FASD is actively working to harmonize research standards in an effort described in more detail by other workshop speakers.

### Practical support tools and emerging innovations

To ensure that clinicians can successfully implement effective screening and diagnostic methods, they need practical support tools. Petrenko said that one promising approach to provide these tools—especially in light of workforce shortages that leave many clinicians with very limited time for professional development—is the Extension of Community Healthcare Outcomes (ECHO), a virtual learning series in which subject matter experts, community providers, and other professionals participate in a case-based learning loop, receive practical recommendations, and build skills [6]. The U.S. Centers for Disease Control and Prevention (CDC) and the American Academy of Pediatrics also have resources to help clinicians build FASD competencies.

Petrenko described several emerging evidence-based technologies to screen for and diagnose FASD at scale that are being developed with support from the Collaborative Initiative on FASD with funding from the National Institute of Alcohol Abuse and Alcoholism (NIAAA). The smartphone application FASD Tree helps clinicians predict what behavioral, cognitive, or physical factors may lead to a diagnosis of FASD [7]. Brain-Online is another virtual screening tool, and Morpheus Q helps providers conduct palpebral fissure, lip, and philtrum measurements [8]. Developers are applying sophisticated new 3D facial imaging tools to detect minute FASD-related facial characteristics, and researchers have also shown how baby teeth can be used to detect PAE [9, 10]. Finally, researchers are exploring how cytokine networks could act as biomolecular markers of PAE and be used to predict developmental delays, which could help to inform interventions and resource allocation [11].

### Influencing change

Research shows that there are interventions that can help to address some of the spectrum of FASD effects, and that these interventions are most impactful when they are to an individual’s neurodevelopmental profile and balance skill building with environmental supports. However, Petrenko said that such interventions have thus far had only a limited impact on the FASD community, largely because of inadequate implementation. In addition, while a variety of FASD interventions that focus on children ages 3 to 12 have been shown to be effective, she suggested that more effort is needed to develop programs for teenagers and adults, both to determine the effectiveness of later interventions and further enhance understanding of the impacts of FASD across the lifespan [12].

To advance FASD-informed care, researchers point to the need for early action and developmentally appropriate treatments that target individual level impairments, as well as relationship-based and trauma-informed care that is attentive to the context in which FASD occurs. In designing programs, it is essential to build collaborative partnerships, empower the community to create culture-centered practices, and work to overcome all barriers, especially stigma.

Closing, Petrenko highlighted additional resources and practical guides for families and professionals, including *Towards Healthy Outcomes for Individuals with FASD*, which provides a roadmap to inform intervention approaches; Families Moving Forward Connect, which offers a virtual continuum of care for patients and provider training; and My Health Coach, an app developed in partnership with the Adult Leadership Collaborative of FASD Changemakers [13–15]. Finally, StrategEase, from the Center for Implementation, offers effective strategies for designing and implementing high-impact provider training and patient-centered programs that deliver the capabilities, motivation, and opportunity needed for success [16].

### Strengthening FASD clinical management and clinical practice guidelines

Delving deeper into some of the strategies used to identify and intervene in PAE and FASD, three speakers highlighted current work and future opportunities to enhance FASD research and clinical management and practice guidelines.

### FASD prevention and clinical guidelines research

The centralized MHS, under the Defense Health Agency, operates more than 400 military hospitals and clinics and serves all branches of the military and their families, with a patient population of 9.6 million people in total, nearly 2 million of whom are children. Ilse Rivera, HJF, discussed an environmental scan of the MHS that USUHS researchers recently conducted across the FASD continuum of care to learn what resources the MHS offers to address alcohol use in general and for pregnant women specifically, as well as what FASD-specific resources exist. Additionally, the effort aimed to identify resource needs, and make recommendations.

The environmental scan revealed that there are clinical practice guidelines, policies, and interventions to screen for and address alcohol and substance use and disorder in pregnancy and pre-pregnancy, which are largely aligned with national recommendations. However, the researchers found that despite these resources, unhealthy alcohol use continues [17]. Notably, researchers found that there are no FASD-specific resources. Together, findings point to a need for FASD-specific screening, support, and services, along with programs to raise awareness of and change the messaging and culture around alcohol use in the military. The MHS has resources for other neurodevelopmental conditions, such as autism, which Rivera suggested could serve as a model when creating FASD-specific supports. Finally, Rivera emphasized the need for comprehensive FASD care to operate across a continuum spanning both mother and child, who need very different types of services, support, and interventions.

### FASD-responsive systems of care

Expanding on her first presentation, Petrenko discussed how the University of Rochester built its FASD Diagnostic and Evaluation Clinic, an example of a FASD-responsive system of care. The process began with a systems scan and needs assessment, similar to the MHS scan Rivera described. This scan revealed a shortage of providers with FASD expertise and a lack of FASD-specific interventions for families. To address these gaps and work toward a FASD-responsive system of care, Petrenko and her team identified a need for community involvement, leadership support, and careful leveraging of existing resources.

The team then studied other disorder-dedicated clinics and gradually assembled the resources to build the FASD Diagnostic and Evaluation Clinic at the Mt. Hope Family Center. The clinic is now fully sustainable, integrated into the pediatric developmental behaviors department, and able to handle 250 new cases a year. Children under age 6 see a single provider, which enables faster diagnosis and access to interventions, while older children either have a single provider or a multidisciplinary care team. Petrenko noted that multidisciplinary care teams bring the benefit of multiple areas of expertise but can also lead to delays in diagnosis or interventions, considerations that must be carefully weighed when determining the best arrangement for each patient.

By successfully optimizing its resources, Petrenko said that the clinic has been able to extend its reach beyond Rochester; improve clinicians’ overall awareness and understanding of FASD; and help more families through support groups, parent resource-sharing networks, and community education programs to reduce stigma surrounding FASD. The clinic also employs ECHO to share its care model more widely, maximizing the impact of its relatively small team by helping to inform other providers’ efforts to set up their own FASD-informed clinics.

### International consensus committee on research classification of FASD

There is no international, interchangeable “gold standard” method to recognize FASD, and researchers and clinicians in different countries use slightly different criteria to define PAE, dysmorphology (especially facial), and characteristics of growth and neurodevelopment. Michael Charness, VA Boston Healthcare System, discussed how this lack of standardization leads to challenges, such as prevalence rates that cannot be compared and research findings that cannot be replicated [18], and described an ongoing effort of the International Consensus Committee on Research Classification of FASD to create a single classification system. Although the committee’s initial efforts are focused on developing a classification system for FASD research, Charness noted that such a system could also be used to harmonize the different clinical diagnosis standards.

To approach its task, the committee is combining the different diagnostic and research databases in each country represented by the committee and analyzing the results for connections between individuals with FASD, PAE, and physical development, including non-sentinel facial features and non-facial dysmorphology measurements that could one day predict neurological development. Once the committee reaches its consensus agreement, it will publish a manuscript on a research classification for FASD, transition researchers to this classification system, and then begin efforts on a consensus process for clinical diagnosis.

### Experiences with FASD: community needs assessment

In a panel discussion, Elizabeth Lee, USUHS; Amanda Woodyard, Military Child Education Coalition; and Paul Patterson, Walter Reed National Military Medical Center, explored how a planned community needs assessment will improve the understanding of MHS members’ experiences caring for individuals with FASD. They discussed some of the unique challenges of a military lifestyle, such as frequent moves, which can influence health risks and also pose barriers for care continuity, along with potential opportunities for the MHS to leverage resources to help families overcome these issues.

### Community needs assessment context and plans

To start the discussion, Lee provided an overview of the goals and preliminary plans for the community needs assessment. The effort’s overarching goal is to understand the lifetime needs and lived experiences of individuals with FASD, caregivers, and healthcare providers across the MHS, spanning the full care continuum from pre-pregnancy through adulthood. Through this effort, researchers hope to identify existing facilitators and barriers to FASD diagnosis, treatment, and services; potential stigmas around FASD screening and diagnosis; attitudes, norms, and beliefs around alcohol use before and during pregnancy; and provider training resources and needs for FASD prevention, diagnosis, and treatment across the care continuum. The main information source will be interviews with key informants and focus groups from three MHS populations: caregivers of children with suspected or confirmed FASD, pregnant people, and interprofessional, interdisciplinary healthcare providers.

Once complete, Lee said that the assessment’s findings will be synthesized into recommendations that address key facets of FASD care in the MHS. Beyond this, the findings can also help inform discussions with families and clinicians in other large healthcare systems; improve EHR analysis and interpretation; inform and refine a telehealth hub-and-spoke model; and be used to identify additional facilitators, barriers, gaps, needs, and recommendations concerning FASD policies, resources, or interventions, Lee said.

Noting that families often need help navigating challenges such as the frequent moves that come with military life, Woodyard stressed the importance of fixing existing service gaps and barriers to access while also broadening awareness of the resources and sources of support that are available. Families who relocate frequently must constantly start over with new healthcare providers. Resources vary widely from place to place, and Woodyard noted that the wait for services can sometimes be longer than the time a family lives in an area, making access nearly impossible. Even within the challenges of the military lifestyle, however, Patterson said that the MHS is well resourced to educate families and providers and ensure that the needs of the most vulnerable community members are addressed. To improve further, he said that lessons can be learned from initiatives that have effectively addressed other neurodiverse conditions and suggested that successful programs should be identified, replicated, and expanded.

### Considerations and strategies for engaging participants

Panelists discussed potential challenges that may be encountered in carrying out the planned FASD community needs assessment and offered suggestions to help ensure the effort is as successful and informative as possible. The community needs assessment requires substantial engagement from the three groups of key informants—caregivers of children, pregnant people, and MHS-connected healthcare providers—each of whom have specific stressors or needs that may influence their participation. To identify eligible families, Patterson suggested tapping into existing processes, such as referrals from pediatric behavioral or neurodevelopmental specialists, who often work in multidisciplinary teams and treat a variety of developmental disorders. He also suggested reaching out to the military-wide Exceptional Family Member Program and Educational and Developmental Intervention Services, as well as primary care or other first-line providers who have access to screening tools, such as the Survey of Well-being in Young Children, to help identify families with FASD.

Once identified, Woodyard said that families will be more likely to participate if the parents trust their healthcare providers and believe they are working for the family’s benefit. In conducting sensitive conversations with potential participants, she said it is important for providers to use neutral, nonjudgmental language to avoid perpetuating stigmas. She added that families may be reluctant to participate in services or interventions if they require family separation, if the families lack adequate resources or support, or if service members fear that participation could have negative consequences for their career. Providers should also consider each military family’s unique dynamics, such as whether the family is new to the military, if there is local family support, or if the pregnant person is a military spouse or in active service, she said.

It is important to recognize that service members, spouses, and children all have different experiences and perspectives, Woodyard noted. Patterson added that researchers must also consider what each group of key informants knows about FASD, how it is diagnosed, and what resources exist for families with FASD, and consider how social stigmas might lead people to omit information about potential PAE. A person’s level of medical literacy, social determinants of health that impact care access, and their willingness to advocate for themselves or their child could also influence their level of participation.

Beyond engaging with families in healthcare settings, Woodyard suggested that some people might be more forthcoming in casual social settings or via social media platforms, which have a broader reach and can support anonymity. It may also be possible to engage families by creating groups tailored to different experiences that encourage sharing perspectives and feedback. Finally, she suggested that providers could collaborate with other trusted adults, such as counselors, chaplains, teachers, or friends, who may be in a better position to listen to the needs of families living with FASD, connect them to support or services, and encourage their participation in the needs assessment.

### Identification of FASD risk factors and diagnoses and modeling FASD pathways of care: research applications for EHRs

Electronic health records (EHRs) represent important sources of data that can shed light on health conditions, their impacts, and strategies for improving care. Three speakers discussed research and surveillance approaches that leverage EHRs and other data sources to identify FASD risk factors, understand current care systems, and find opportunities to improve care.

### Research applications for military EHRs

In addition to the MHS environmental scan, the FASD Prevention and Clinical Guidelines Research initiative plans to conduct a population health assessment and map the MHS pathways of care across the entire care continuum. Lee described the goals and scope of these efforts.

The overarching goal of the population health assessment is to understand the prevalence burden of alcohol use and disorders prior to and during pregnancy, prenatal alcohol exposure, and FASD-related diagnoses. Researchers plan to accomplish this through a retrospective analysis of secondary data from 2010 onward, drawing data from the centralized MHS data repository, which includes administrative, demographic, and claims-based data. The study population includes TRICARE beneficiaries of childbearing age with at least one pregnancy, along with all MHS beneficiaries with one or more FASD-related diagnoses.

The first priority of the population health assessment is to determine the epidemiology of FASD-related diagnoses across the FASD continuum of care, accounting for sociodemographic factors. The team will develop risk-based criteria to identify children with suspected or confirmed FASD, identify and describe the constellation of FASD-related diagnoses, and calculate annual adjusted rates of FASD-related diagnoses to explore trends over time. The second priority is to identify characteristics and risk factors associated with FASD-related diagnoses and develop and test a predictive analytic tool that identifies individuals with undiagnosed FASD for screening.

In a separate effort, researchers are working to map MHS care pathways for FASD. Pathways of care are comprised by the details involved in identifying, referring, evaluating, diagnosing, and providing care to patients. To evaluate care pathways and their outcomes for FASD-related conditions within the MHS, researchers will first investigate questions such as who provides the care, what specialties are involved, what care is actually provided, and how frequent the clinical interactions are. Next, researchers will identify the facilitators and barriers of care along these pathways, explore the characteristics of the children who are receiving this care, and identify how certain factors may be used to predict outcomes such as the average age at related diagnosis, time to diagnosis, and time to first specialty visit.

### FASD prevalence and risk factors

Philip May, University of North Carolina at Chapel Hill, discussed research insights into FASD prevalence and its risk factors. A conservative estimate of the FASD prevalence rate in the United States is 3 to 10%, with the mean at 6.5% [19]. The most influential known risk factor is prenatal alcohol exposure [20]. Maternal risk factors for FASD encompass a woman’s general physical and mental health; the type, quantity, frequency, and gestational timing of alcohol use; and social norms and practices [21]. Paternal behavior and exposures also influence a child’s genetics and epigenetics and can also enable or discourage maternal alcohol consumption [22]. Several other risk factors also impact the early development of children with FASD, such as the prevalence of alcohol in breast milk, home stability and security, and educational opportunities [23]. May emphasized that positive reinforcers of child development are strong predictors of successful outcomes for children with FASD.

### Leveraging multiple data sources for surveillance of children with FASD

Public health surveillance of children with FASD is a priority for the CDC, but just as there is no standard research definition for FASD, there is also no standard surveillance case definition. Shin Y. Kim, CDC, described how I-FASD, a public health surveillance partnership involving Emory University and the Minnesota Department of Public Health, is examining the feasibility of using existing clinical healthcare data, healthcare claims databases, and caregiver-reported estimates to inform surveillance efforts.

First, investigators are working to identify relevant data sources and elements within health records to understand where information lives and also determine whether they use common procedure, diagnostic, or billing codes. Kim noted that the MATernaL and INfant Clinical NetworK (MAT-LINK), which has a focus on medication for opioid use disorder, has been a useful model to help the team understand what relevant data elements are available in medical records.

Next, investigators will characterize the availability and quality of data, including for children who have been evaluated for but not diagnosed with FASD, and then conduct qualitative interviews with clinicians and data stewards to identify the benefits and drawbacks of using this data for FASD surveillance. Finally, the team will conduct qualitative interviews with individuals with FASD, their families, and clinicians to illuminate the processes and pathways involved in the continuum of care, from referrals to communication to diagnosis to care access or delays.

Ultimately, the findings may help inform the development of surveillance activities relevant to assessing prenatal exposures, adverse birth outcomes, and outcomes throughout the lifespan. Through this effort, investigators aim to improve understanding of the data sources and variables that are best suited for identifying children with FASD; illuminate the strengths, challenges, and opportunities for leveraging clinical data to conduct public health surveillance; and contribute useful knowledge about FASD referral, evaluation, and diagnostic processes.

### Patient experiences and perspectives on clinical management of FASD

To bring into the discussion the voices and lived experiences of individuals with FASD, Jenn Wisdahl, FASD United, introduced four speakers as “the people behind the statistics.” These panelists included two members of the Adult Leadership Committee of the patient advocacy organization FASD Changemakers, CJ Lutke and Justin Shepherd; self-advocate Brenna French; and Julia Rivera, U.S. Air Force (Ret.), mother of a child with FASD.

Lutke said that she was diagnosed at birth with what doctors then called fetal alcohol syndrome (FAS). Her parents were told that the situation was “hopeless,” but they disagreed and fought to change the perception of what it means to have FAS. Living with FAS/FASD can be very difficult, Lutke said, but early diagnosis and family support can make an enormous difference. She stressed that preventing FASD—and ensuring that a diagnosis of FASD does not define a person—requires accurate diagnoses, appropriate interventions, family navigators and advocates, access to services, and support groups to address both FASD and its secondary characteristics. “There are things I cannot and will never be able to do,” Lutke stated. “But, I have more things that I *can* do, and a couple things I can do really, really well. This is the story for most people with FAS.”

Unlike Lutke, Shepherd was not diagnosed with FASD until adulthood. He was unaware of his condition until he started filming a documentary about individuals with FASD and recognized himself and his behaviors in the interviews he was conducting. He was then diagnosed with an alcohol-related neurodevelopmental disorder and alcohol-related birth defects, both of which are part of FASD’s large, confusing spectrum that makes misdiagnosis common. He said that organizations like the FASD Changemakers Adult Leadership Committee do critical work and make it easier to see people as the individuals they are through sharing stories, raising awareness, reducing stigma, and spurring progress to improve the lives of people with FASD. To prevent FASD, he said it is important to avoid blaming mothers and focus instead on creating society-wide support and education for pregnant people.

French was 12 years old when she was diagnosed with FASD. She said her diagnosis came as a relief, as it not only explained why she felt so different from her peers and helped her accept herself the way she was, but also led her to access services such as music therapy, recreational therapy, and behavioral therapy that have helped her flourish. Her diagnosis also improved her relationships with teachers and extended family, who were able to better understand her in the context of her condition and its effects. Now a teenager, French advocates for greater FASD awareness, along with understanding and compassion for those with FASD. “My diagnosis opened doors to support and services that allowed me to thrive, develop essential skills, and achieve things I have never thought possible,” she said. “I've learned that having a disability doesn't define who I am or what I can achieve.”

Rivera shared her experience as the mother of an adopted child who was diagnosed with FASD at age 14. Until this diagnosis, Rivera said her family had seen multiple medical specialists and tried various programs and medications, some of which were quite extreme. Yet, her son still struggled, especially with what she now knows are the secondary characteristics of FASD, which can involve problems in school, at work, or with substance abuse. Finally receiving a diagnosis of FASD, which Rivera said was likely needlessly delayed due to a lack of provider awareness of FASD, was pivotal for her family by enabling them to better understand their son and address the challenging secondary characteristics of his condition.

Shepherd shared that his diagnosis has taught him to self-advocate, even to the extent that he educates his own healthcare providers about the condition when necessary. He said that when he was younger, less was known about how to help children with behavioral issues or disorders, but he always knew something about him was different. Noting that he missed out on the specialized attention and understanding that might have helped him if he had been diagnosed with FASD earlier, Shepherd said that increasing FASD awareness is important to improving understanding and accommodation and help children with FASD feel more accepted and supported.

Even though she was diagnosed at birth, Lutke said that, like Shepherd, she also felt unsupported and misunderstood because the knowledge of FASD was so limited 30 years ago. She said she is grateful that schools and teachers today are much more willing to make the effort to understand student behavior and are less likely to label children as “bad kids.” Rivera agreed that for people with FASD, understanding your condition is crucial to accepting yourself.

### Delivering a telehealth hub and spoke intervention for FASD

In addition to increasing understanding of FASD and informing clinical guidelines for prevention and care, Eric Flake, HJF, and Jeanmarie Rey, USUHS, stated that a major goal of the FASD Prevention and Clinical Guidelines Research initiative is to implement and evaluate a telehealth hub-and-spoke model for FASD diagnosis and care within the MHS. As many workshop speakers noted, it is challenging to pinpoint an exact FASD etiology and characterize its unique subset of neurodevelopmental impacts. However, accurate diagnosis is critical to help those with FASD forge an identity and create a treatment pathway for long-term care. It is therefore important for clinicians, researchers, and advocates to collaborate to identify impacts, measures, and solutions that can be used to make a diagnosis of FASD, craft meaningful interventions, and share them widely via a telehealth hub-and-spoke model.

Flake and Rey introduced three panelists who discussed multiple aspects of designing and implementing a successful telehealth hub-and-spoke model for FASD-responsive systems of care, including the benefits of telehealth, challenges of diagnosing FASD, addressing stigmas, and the importance of screening and partnerships. The panelists were Hope Finkelstein, Alaska Department of Health; Douglas Waite, Bronxcare Health System; and Vincent C. Smith, Boston Medical Center.

### Context on key needs

To provide context for their discussion of a telehealth hub-and-spoke model, panelists first offered opening remarks on key needs in FASD diagnosis and care. Finkelstein said that successful public health initiatives can create systems changes for the benefit of individuals, families, and communities. She suggested that a more holistic, comprehensive approach to creating lasting change will require adding another focus; beyond diagnosis (which remains critical), she said that it is also important to uncover more knowledge about the larger ecology of issues related to PAE, such as trauma, equity, and social and ecological determinants of health.

Waite agreed that accurate diagnosis of FASD remains challenging with today’s multidisciplinary models. An FASD diagnosis, made as early as possible in a child’s life, serves as a vital platform for explaining to families what strengths and challenges to expect as their child grows. In addition, he said that additional training for primary care providers to perform the detailed PAE screening essential to enabling early diagnosis. Adding that diagnosis is only the first step toward stable access to consistent, effective interventions, Waite said that it will also be critical for the MHS to ensure continuity of care in light of the frequent moves military families make.

Smith brought his perspective as the medical director for the American Academy of Pediatrics Fetal Alcohol Spectrum Disorders Program. At Boston Medical Center, he is also involved in SAFEST Choice, an FASD learning collaborative that aims to raise awareness of FASD, especially in the education system. In addition to helping families to access the support they need at home, Smith said that training teachers to recognize and understand FASD can enable them to see past the classroom challenges children with FASD face and help them maximize their potential.

### The benefits of telehealth

Panelists said that telehealth represents an important opportunity to increase clinicians’ capacity to diagnose and care for individuals with FASD and help more families reach clinicians who are knowledgeable about the condition. Waite said that telehealth platforms can be useful for training a wide range of professionals who work with children—including medical residents, pediatricians, psychiatrists, social workers, and juvenile justice staff—to spread awareness of the need for PAE screening, expand understanding of FASD diagnostic criteria, and increase the number of accurate FASD diagnoses. Telehealth can also be used to design and share post-diagnosis family support strategies.

Smith agreed that telehealth training—particularly training delivered via ECHO—could expand diagnostic capabilities around the country by encouraging a standard curriculum to ensure that participants gain a baseline level of knowledge, using real-life cases and issues as co-learning models, and enabling peer-to-peer learning and collaboration. He added that a major strength of the ECHO platform is that it is not geographically limited and does not rely on local resources. Beyond ECHO, more approaches to address post-diagnostic services for families with FASD. He also pointed to the need to address key barriers to care access, such as policies that prohibit clinicians from delivering telehealth services across state lines.

The COVID-19 pandemic greatly increased people’s comfort level with telehealth across society, Finkelstein noted, and as a result, many people are now well-versed in the use of virtual meetings to share knowledge and expertise, raise awareness, increase engagement, and build competencies and capabilities. To maximize the positive outcomes from telehealth hub-and-spoke interventions for FASD, she posited that such interventions must be specifically targeted to FASD characteristics, include appropriate reimbursements, ensure continuity of care, and incorporate ways to measure health, not just sickness.

Smith agreed that continuity of care is crucial, particularly given the frequent location changes military families experience. He suggested that using telehealth to improve communication between providers could help improve continuity of care and facilitate more holistic interventions.

### Challenges with diagnosis

Panelists discussed how a telehealth hub-and-spoke model could potentially help to address one of the key gaps in FASD care: the need for timely and accurate diagnoses. Smith stated that diagnosing FASD is very difficult without either a documented history of prenatal alcohol exposure or the distinctive facial characteristics that are found in only about 10% of individuals diagnosed with FASD. An objective test or biomarker to support FASD diagnosis without PAE history would be incredibly helpful. It would also be helpful to learn how many individuals with PAE have an FASD diagnosis and track their health over their lifetime. Knowing a person’s PAE details, as early as possible, enables tailored interventions.

Waite agreed, adding that diagnosing developmental delays and differentiating their causes, especially at very young ages, is both challenging and subjective, yet accurate diagnoses are essential for receiving appropriate interventions.

Finkelstein cautioned that while a hub-and-spoke model could help to improve clinicians’ knowledge of FASD and increase their confidence in making diagnoses, it is important not to overlook the social and environmental factors that can influence FASD diagnosis and care, such as provider biases or a lack of family support systems. She said that a more holistic, strengths-based care pathway must incorporate these factors to recognize strengths and promote health, provide appropriate reimbursement codes for providers, reflect the range of neurodevelopmental disorders under the FASD umbrella, and create a multi-tiered model of PAE that benefits more people.

### Addressing stigmas

Throughout the workshop, participants stressed the importance of addressing the shame and stigmas associated with PAE and FASD, which can pose a significant barrier to getting appropriate diagnoses and care. Smith said that talking publicly and frankly about FASD is key to removing stigmas. If a celebrity discussed their experience, the way Magic Johnson shared his HIV diagnosis in 1991, for example, it could help to normalize the condition and create space for public conversation. Stigmas can prevent people from being honest about alcohol use during pregnancy and participating in interventions, especially when there are potential or perceived negative consequences from an official FASD diagnosis, for example, fear of legal repercussions for PAE. Smith said that the existing punitive model must be replaced by one that is caring and treatment-focused, so that families with FASD can focus on their health.

Waite added that adjusting terminology, such as referring to “neurotoxin exposure” instead of “alcohol exposure,” can help to remove some of the blame by putting alcohol in a category alongside other neurotoxins, like pesticides, that are known to have negative effects on brain development. He suggested that the term FASD itself could pose a barrier to getting more children diagnosed, particularly in the military setting.

Rey added that provider awareness and training can help to minimize biases and negative consequences, and added that the FASD Prevention and Clinical Guidelines Research initiative will identify solutions to the challenges and barriers military families face, such as frequent moves, partly by identifying and replicating successful programs in the hub-and-spoke model. Another participant suggested using ECHO for this purpose and emphasized the need to go beyond identifying barriers to design effective solutions supported by training and resources.

### The importance of screening and partnerships

Waite suggested that a relatively quick and easy change to demonstrate the impact of a hub-and-spoke model would be to disseminate training for military pediatricians, family practitioners, and other providers to increase the number of people who are screened for PAE to support future FASD diagnosis. More screening increases overall awareness and plants a seed in patients’ minds that can lead to a later recognition and diagnosis. Waite added that children who have been exposed to opioids should also be screened for PAE, and Smith said that obstetricians and gynecologists could be included in the list of providers who should receive training on prevention and screening for PAE.

Rey noted that partnerships will be essential to accomplishing these large, system-wide goals, and Finkelstein agreed that successful partnerships that pool multiple strengths can enable significant achievements. For example, a community-wide project in Kenai, Alaska, provided clinics, school districts, parenting groups, and tribal communities with enhanced FASD education, which led to the establishment of more partnerships, an increase in requests for services, and a reduction in school disciplinary actions.

Waite stated that partnering with schools can be especially helpful for identifying potential children with FASD and starting early interventions, which also require trusted relationships with providers and continuity of care. Telemedicine can help with continuity of care, but he emphasized that training for schools, especially schools on or near military bases, is also important to increase awareness, reduce stigmas, and enable more people to benefit from interventions.

### Federal agency updates

For the workshop’s final session, Donaldson invited representatives from federal agencies to discuss current and planned federal activities aimed at advancing the understanding of FASD and improving care for individuals and families.

### National institute of alcohol abuse and alcoholism

Bill Dunty shared context and program updates from NIAAA, an institute of the National Institutes of Health whose mission is to generate and disseminate knowledge about alcohol’s effects on people’s health in order to improve lifelong diagnosis, prevention, and treatment of alcohol-related problems. Dunty said that research to advance understanding of FASD etiology, prevention, interventions, and diagnosis is an agency priority, and added that the institute also financially supports other research centers that study PAE.

While progress has been made since FASD was officially recognized 50 years ago, Dunty emphasized that there is much more work to do. It is estimated that 1 in 10 pregnant women in the U.S. consumes alcohol, and of those, 1 in 5 use multiple substances [24, 25]. Key challenges NIAAA programs are focused around include preventing alcohol-exposed pregnancies, improving early identification of children with FASD, developing and implementing interventions at all ages, understanding how PAE impacts chronic disease risk, reducing the stigma around FASD that hinders prevention efforts, addressing barriers to improving FASD awareness, and studying the impact of PAE on placenta and gut microbiome function. NIAAA also puts out notices of funding opportunities to encourage research and participates in NIH-wide pediatric initiatives, such as the Adolescent Brain and Cognitive Development Study and the Healthy Brain and Child Development Study, Dunty noted.

### Interagency coordinating committee on fetal alcohol spectrum disorders

Tatiana Balachova, NIAAA, who serves on the Interagency Coordinating Committee on Fetal Alcohol Spectrum Disorders (ICCFASD), provided an overview of ICCFASD efforts. Launched in 1996 and supported and administered by NIAAA, the ICCFASD works to improve communication, cooperation, and collaboration among federal agencies that address issues related to FASD. The challenges addressed as part of this effort reflect the complex web of issues that impact those with FASD as well as the diversity of the missions and goals of the participating agencies, which include the Office of the Assistant Secretary for Planning and Evaluation (ASPE), the Administration for Children and Families (ACF), CDC, Health Resources and Services Administration (HRSA) the Indian Health Service (IHS), the NIAA, Eunice Kennedy Shriver National Institute of Child Health and Human Development (NICHD), the Centers for Medicare & Medicaid Services (CMS), the National Institute on Drug Abuse (NIDA), the National Institute of Mental Health (NIMH), Substance Abuse and Mental Health Services Administration (SAMHSA), and the Administration for Community Living (ACL). The ACL is the newly joined agency, whose mission is to support the independence, well-being, and health of older adults and people with disabilities.

Balachova highlighted two new CMS programs that are particularly relevant to FASD. The first delivers screening and services to pregnant opioid users, who may also consume alcohol. The second focuses on improving integrated case management services for children with multiple conditions including FASD.

The ICCFASD also hosts yearly public meetings, convenes working groups to address specialized topics, shares funding opportunities and information about specific programs, and highlights new programs and ideas. Work advanced and disseminated by the interagency coordinating committee has included, for example, a survey of community assistance programs for individuals with FASD, FASD United’s new Family Navigator Program, and new efforts to better incorporate the perspectives of people and families with FASD. In addition, the ICCFASD encourages agency researchers to examine emerging issues, create stronger prevention tools, consider the impact of mental health and stress on behavior during pregnancy, and address evidence gaps in PAE and FASD screening.

### U.S. centers for disease control and prevention

Elizabeth Parra Dang shared updates on CDC efforts. Despite evidence of its efficacy, studies show that alcohol screening and brief intervention (SBI) is not part of routine prenatal care, and only 1 in 4 postpartum women recall being advised about the risks of alcohol use [26, 27]. Many clinicians do not feel confident conducting alcohol SBI, and many pregnant people who report substance use engage in multiple substances, not just alcohol [25, 28].

To increase the use of prenatal alcohol SBIs, the CDC has worked to enhance provider training programs, presented at national and international conferences, investigated the barriers that prevent local healthcare providers and grassroots organizations from providing multiple-substance prenatal and postpartum screening, and clarified messaging and recommendations. To convene relevant organizations, the CDC also created the FASD National Partner Network, a collaborative framework of provider, non-clinical professional, and family support organizations that work to improve the lives of families with FASD. In addition, the CDC recently released new materials to improve communication between providers and pregnant people both in general and specifically about the risks of alcohol during pregnancy. These materials will be disseminated through the FASD National Partner Network as well as within EHRs and patient portals.

Dang said that future CDC projects will collect and integrate more of the lived experience of families with FASD into messaging and weave these resources into larger conversations about other types of substance use during pregnancy.

### U.S. department of health and human services

Sharon Newburg-Rinn spoke about FASD-related initiatives of the Children’s Bureau (CB) of the U.S. Department of Health and Human Services (HHS). The work of the CB, within the HHS Administration for Children and Families (ACF), often intersects with FASD, as it collects data on children in contact with the Child Welfare System, a disproportionate number of whom have a history of prenatal alcohol or drug exposure. Unfortunately, FASD is often misdiagnosed or undiagnosed in this population. Newburg-Rinn said that better strategies are needed to screen and, as applicable, diagnose and refer welfare system-connected children with FASD. While ideally children with FASD should be able to access necessary services within the home, she noted that the military must abide by relevant state rules, which may remove children from a parent who abuses substances prenatally and place them in state or military foster homes.

The CB is also developing resources for the Child Welfare League of America that reflect common misperceptions about prenatal alcohol use and its detrimental effects, with assistance from tribal representatives, an advisory committee of national FASD experts, and researchers sensitive to the ways in which race and ethnicity can intersect with trust and participation in research and healthcare. The CB is also collaborating with the National Center on Substance Abuse in Child Welfare and Head Start leadership on future plans.

Finally, Newburg-Rinn said that when it was tasked by the White House to develop an idea with a positive impact but without requiring new funds or laws, the ACF created an “Information Memorandum” for all ACF Regional Offices and State Child Welfare Agencies. The memo underscores the importance of identifying children with FASD, providing appropriate services, correcting misunderstandings or biases, and avoiding blaming or scaring parents.

### Health resources and services administration

The mission of the Maternal and Child Health Bureau (MCHB) of the HRSA is to improve the health and well-being of the nation’s mothers, children, and families through access, equity, capacity, and impact. Sonsy Fermín described how MCHB’s Supporting Fetal Alcohol Spectrum Disorders Screening & Intervention (SFASDSI) Program uses ECHO to train multiple diverse provider cohorts at community health centers and tribal clinics across the U.S. to screen and provide interventions or referrals to pregnant people, screen children and adolescents for PAE, and connect both groups with FASD services and care.

SFASDSI has core FASD-focused, interactive content modules and specific content to engage prenatal and pediatric providers, as well as additional educational content and mixed-method evaluations to ensure participant satisfaction and an overall understanding of the material. Fermín said that the initial successes of the program have led to multiple extensions, with more expected.

## Closing reflections

To close the workshop, Koehlmoos and Donaldson thanked attendees and speakers for sharing their ideas and expertise in a productive second workshop, and extended their gratitude to all participants in the broader FASD community for contributing their knowledge and experiences. As the FASD Prevention and Clinical Guidelines Research initiative continues to make progress on its many lines of effort—the MHS environmental scan, community needs assessment, population health assessment and EHR analyses, and telehealth hub-and-spoke model—and as other related research projects and interventions described at the workshop continue to move forward, they expressed optimism that the community will be closer to the goal of improving the lives of military families with FASD.

### Future research

The presentations and panel discussions presented during this workshop have provided valuable input with which to shape the remaining phases of the FASD Prevention and Clinical Guidelines Research project. Following this year’s workshop, work has begun on the community needs assessment, the identification of FASD risk factors and diagnoses, and the tele-education phases of the project. We look forward to disseminating any findings from these efforts at the next workshop.

## Data Availability

All data is contained within the workshop report.

## Appendix

### Workshop Attendees

**Table:**

Organization Type	Organization	Participant
Government	Alaska Department of Health	Hope Finkelstein, MA
	Centers for Disease Control and Prevention	Elizabeth Parra Dang, MPH
		Shin Kim, MPH
	Health Resources and Services Administration	Sonsy Fermín, MSW
	HHS, Children’s Bureau, Administration for Children & Families (ACF)	Sharon Newburg-Rinn, PhD
	National Institute on Alcohol Abuse and Alcoholism	Tatiana Balachova, PhD
		Bill Dunty, PhD
	Uniformed Services University of the Health Sciences	Paul Crawford, MD
		Joshua Gray, PhD, Co-I
		Dean Lynette Hamlin, PhD, CNM, Co-PI
		COL Patrick Hickey, USA, MD, MPH
		Kristin Kepler, DNP, WHNP-BC
		Tracey Koehlmoos, PhD, MHA, PI
		Elizabeth Lee, DrPH, MPH, Co-I
		CDR Monica Lutgendorf, USN, Co-I
		COL Dana Nguyen, USA, MD
		COL Cadet Nyland, USAF, MD
		LtCol, Jeanmarie Rey, USAF, MD, Co-I
	South Carolina Department of Social Services	Michelle Cunningham, BSN
	VA Boston Health Care	Michael Charness, MD
	Walter Reed National Military Medical Center	LTC Paul Patterson, MD, PhD
Non-profit organizations	Arkansas None for Nine	David Deere, MSW, MTh
		Shaneca Smith, BSM. RM, CNOR(E)
	Alaska Center for FASD	Michael Jeffery, JD
		Teri Tibbett, MFA
	Dream Acres	Kathryn Meinhardt, BSW
	FASCETS	Natalie Brassard, RDN
		Sil Pienovi
	FASD Focus NW	Joanne Sparrow, PhD
	FASD Maine	Constance Mazelsky, BA
	FASD Network of Northern California	Vitka Eisen, MSW, Ed.D
		Barbara Hansen, MEd
		Rachel Sing
	FASD Network of Southern California	Annette Kunzman, MBA
		Janis Reid, MSW, LCSW
	FASD North Dakota	Carl Young, MS
	FASD United	Emma Baldwin, BS
		Elizabeth Barlet, MD
		Susan Carlson, BA
		Tom Donaldson, BA, Co-I
		Susan Elsworth, BA
		Katie Erdman, BA
		Heather French, BA
		Casidee Gonzales, BA
		Andy Kachor, BA
		Chris Melfi, BA
		Jenn Wisdahl, BA, Co-!
	Formed Families Forward	Kelly Henderson, BA
		Stacia Stribling, PhD
	Indiana Alliance on Prenatal Substance Exposure	Jackie Franks, MPH, CHES, CPS
		Emelia Ottinger, BSW
	Illuminate Colorado	Marilyn Fausset, MS
		Lex Loutzenhiser, MLS
		Shira Shump, M.Ed
		Jade Woodard, MPA
	Kansas FASD Support Network	Holly Bane, BS
		Kathryn White
	MCFARES (Michigan Coalition for Fetal Alcohol Resources, Education and Support)	Mindy Barela, MA
		Emily Rusnak, Ph.D., CCC-SLP
	Military Child Education Coalition	Amanda Woodyard, EdD
	NACCHO	Harpur Schwartz, MSPH, CHES, CLC
	NCFASD Informed	Terry Henson
		Kathy Hotelling, PhD, ABPP
	North American Council on Adoptable Children	Barb Clark, BIS
	Proof Alliance	Kendra Gludt, MPH
	Texas FASD Network	Laura Bousquet
		Angelle Gremillion
		Katie King, RN
		Lyn McMurry
		Siara Rouzer, PhD
	The Florida Center	Tamra Cajo, PhD
		Kathryn Shea, MSW
		Jennifer Werden, MSW
	The Papillion Center	Erin Goodman, MA
		Chris Troutt, LMFT
		Ashley Turner
Companies and consultancies	Guided by Guida	Guida Brown, MS
	Nurturing Family Counseling	Kelli Justusson, LPC
	United States Drug Testing Laboratory, Inc	Aileen Baldwin, PhD, MPH
	Way to Grow	Barbara Luborsky, OTR/L
Patients and families	Self/no affiliation	Brenna French, BA
		Julia Rivera, Col USAF, Judge Advocate General
		Justin Shepherd
	FASD ALC Changemakers	Catherine (CJ) Lutke
		Janice Lutke
Academic research and health care organizations	Boston University	Rachel Sayko Adams, PhD, MPH
	Bronxcare Health System	Michelle Kuhn, PhD
	Childrens National	Kazue Hashimoto-Torii, PhD
	Henry M. Jackson Center for the Advancement of Military Medicine, supporting the Center for Health Services Research at USUHS	Cathaleen Madsen, PhD
		Ilse Rivera, MPH
		Zoe Solomon, MPH
	OhioGuidestone DoubleARC Center for FASD	Brianna Megyesi, MSW, LSW
	University of North Carolina at Chapel Hill	Philip A. May, PhD
	University of Rochester	Christie Petrenko, PhD
		Jenna Hiller
Organization not specified		Sean Patrick Bousquet
		Megan Catherwood
		Jean Clarkson
		Bruno Coelho
		Denise Cunha
		Mary Donahee-Rader
		Carmen Grinstead
		Todd Hirschey
		Ryan Jolly
		Faith Carria Joy
		Liz McHugh
		Anne Munson
		Aaron Myers
		Kathryn Pitrat
		Erika Rice
		Heather Royer
		Teena Saini
		Lenette Serlo
		Rebecca Tillou
		Masaaki Torii
		Kelly Warfield
		William Wolfe

## Abbreviations

FASD: Fetal alcohol spectrum disorders
USUHS: Uniformed Services University of the Health Sciences
DoD: Department of Defense
MHS: Military Health System
CHSR: Center for Health Services Research
HSR: Health Services Research
HER: Electronic health records
PAE: Prenatal alcohol exposure
CDC: Centers for Disease Control and Prevention
NIAAA: National Institute of Alcohol Abuse and Alcoholism
HJF: Henry M. Jackson Foundation for the Advancement of Military Medicine, Inc.
CMS: Centers for Medicare & Medicaid Services
ICCFASD: Interagency Coordinating Committee on Fetal Alcohol Spectrum Disorders
SBI: Alcohol screening and brief intervention
CB: Children’s Bureau
HHS: U.S. Department of Health and Human Services
ACF: Administration for Children and Families
MCHB: Maternal and Child Health Bureau
SFASDI: Supporting Fetal Alcohol Spectrum Disorders Screening & Interventions
